# Epidemiology of first-time major lower extremity amputations– A Danish Nationwide cohort study from 2010 to 2021

**DOI:** 10.1007/s10654-025-01210-3

**Published:** 2025-03-12

**Authors:** Anna Trier Heiberg Brix, Katrine Hass Rubin, Tine Nymark, Hagen Schmal, Martin Lindberg-Larsen

**Affiliations:** 1 Department of Orthopedic Surgery and Traumatology, Odense University Hospital, Odense, Denmark; 2 Department of Clinical Research, University of Southern Denmark, Odense, Denmark; 3 OPEN - Open Patient Data Explorative Network, Odense University Hospital, University of Southern Denmark, Odense, Denmark; 4 Department of Orthopedics and Traumatology, University Medical Center Freiburg, Freiburg, Germany

**Keywords:** Major lower extremity amputations, Register-based, Trends, Demographics, Femoral amputation, Incidence

## Abstract

**Background and Aim:**

Major lower extremity amputations (MLEA) are common procedures. Potential changes in surgical strategy and patient characteristics over time have not been described previously. The aim of this study was to investigate the incidence rates and surgical strategies of first-time MLEAs over time from 2010 to 2021. Furthermore, to describe patient demographics, and their changes in the same period.

**Methods:**

This is an observational nationwide register study including all first-time MLEAs performed in patients ≥ 18 years from 2010 to 2021, with data from the Danish National Patient Register.

**Results:**

A total of 12,672 first-time MLEA patients were identified from 2010 to 2021. The annual number of first-time MLEAs each year was unchanged at approx. 1000 annually during the study period. In 2021 the total incidence was 21.3/100,000 inhabitants and the total adjusted incidence rate decreased by 2.3% (95% CI 1.8–2.8) per year. The adjusted frequency of transfemoral amputations increased significantly with 10.9% each year confidence interval (CI) (9.7–12.0), whereas knee disarticulation(-19.4%/year CI (-22.2- -16.5)) and transtibial amputation (-7.3%/year CI (-8.5- -6.1)) significantly decreased. The frequency of primary hip disarticulations were stable throughout the study period (p-value 0.06). When analyzing patient comorbidity profiles we found no major changes over time. When statistically testing for time trends, only dyslipidemia (5.7%/year CI (4.5–7.1)), renal insufficiency (1.8%/year CI(0.2–3.3), peripheral artrial disease (-9.3%/year CI (-10.8- -7.7)) and cardiovascular disease (-3.4%/year CI(-4.6- -2.1)) showed a significant time trend in the study period.

**Conclusions:**

We observed a decreasing incidence of first-time MLEA in Denmark and a shift towards increased use of transfemoral amputations as initial MLEA level. Investigation of the comorbidity profile of MLEA patients revealed some time trend changes during the study period, but with limited clinical relevance. Hence, the observed prominent shift towards a more proximal first time amputation level in Denmark did not seem to be associated with an altered comorbidity profile of these patients. Whether the change in surgical strategy is to the benefit of the patients should be investigated further.

**Supplementary Information:**

The online version contains supplementary material available at 10.1007/s10654-025-01210-3.

## Background

Major lower extremity amputations (MLEAs) can be divided into hip disarticulation (HD), transfemoral amputation (TFA), knee disarticulation (KD), and transtibial amputation (TTA) [1]. A worldwide decrease in the incidence of MLEAs has been reported, although with geographical differences [2–5]. In the western world, 90% of amputations are estimated to be due to underlying vascular disease [6]. Only a few studies have examined surgical strategies in MLEA surgery in detail. Walter et al. reported an increasing trend in TFA and TTA procedures, and a major decline in KD procedures from 2015 to 2019 in Germany [5]. In general, surgical strategies are rarely described in detail, but grouped as either major/minor or above knee/below knee, with no exact consensus on definition [7]. The burden of co-morbidity is substantial in MLEA patients and high frequencies of peripheral arterial disease (PAD) (80%), hypertension (51–83%), diabetes (29-39%), and renal insufficiency (14%) have been reported [3, 8, 9].

Endovascular procedures have increased since 2002. In two studies with data from 1997 to 2014 and 2016–2019 the number of major amputations after revascularization and the number of prior revascularization procedures in patients with PAD or diabetes, are reported stable [3, 10].

The aim of this study was to investigate the incidence rates and surgical strategies of first-time MLEAs over time from 2010 to 2021. Furthermore, to describe patient demographics, and their changes in the same period.

## Method section

This is an observational cohort study with data from the Danish Nationwide Health registers. The RECORD guidelines for reporting routinely collected observational data were followed [11].

### Data sources

Data on all first-time MLEA procedures from January 1, 2010, to December 31, 2021, was obtained from the Danish National Patient Register (DNPR). DNPR contains data on all Danish hospitalizations, including ICD-10 diagnoses and NOMESCO surgical procedures since 1977, with an estimated completeness of > 99% [12]. Identification of cases and linkage between registers was possible with the unique social security number, a 10-digit personal number assigned to all persons living in Denmark [13]. Data from the DNPR include information on gender, amputation procedure, and ICD-10 diagnosis codes.

Data from The Danish National Prescription Database (DNPD) was used to enhance the validity of diabetes, hypertension and dyslipidemia diagnosis [8, 14]. The DNPD contains data on all reimbursed prescriptions from 1995 classified by Anatomical Therapeutic Chemical (ATC) codes [15].

### Study population

**Fig. 1:**
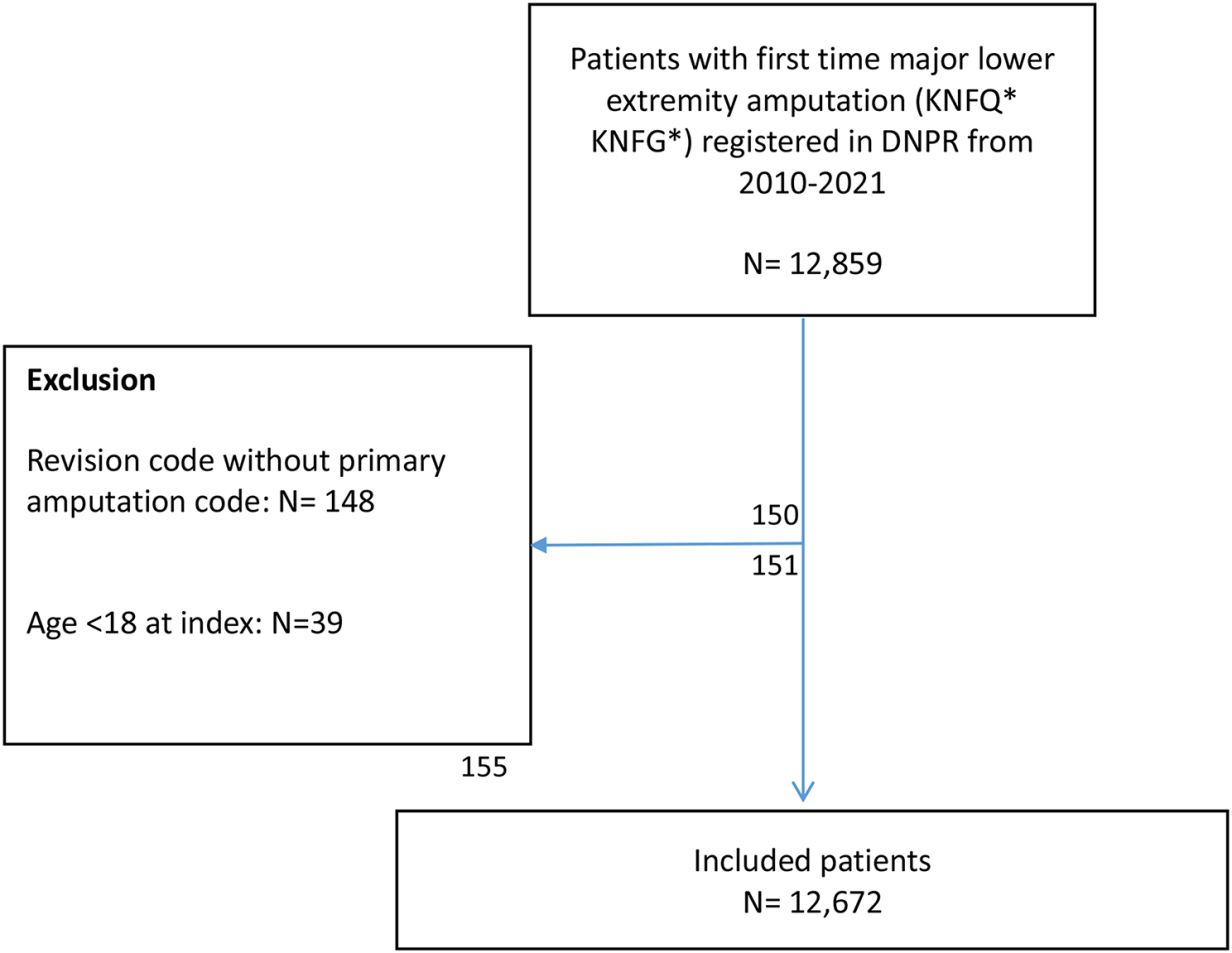
– Flowchart of included cases. DNPR: Danish National patient registry

The population includes all patients ≥ 18 years old undergoing a first-time primary MLEA procedure in Denmark from January 1, 2010, to December 31, 2021, defined through procedure codes (amputation on thigh/hip (KNFQ*) and amputation on lower leg/knee (KNGQ*) the DNPR (Fig. 1). To ensure that the index MLEA was a first-time MLEA conducted in the study period (2010–2021), all patients with an amputation procedure code; amputation on thigh/hip (KNFQ*) and/or amputation on lower leg/knee (KNGQ*) from 1996 to 2009 was excluded from the raw data set prior our obtainment (washout). Due to applied data protection rules in Denmark, The Danish Health Data Authority conducted this exclusion of prior cases. An overview of included cases is presented in Fig. 1.

Studies based on the same raw data, but with different study populations and aims, has been conducted [16, 17].

### Definitions

Hip disarticulation, transfemoral amputations, knee disarticulations, and transtibial amputations are classified as Major Lower Extremity Amputations (MLEA). An amputation through the ankle joint (Syme’s procedure) was not included as a major amputation, but as a minor amputation. Below/through-knee amputation (BKA) consists of transtibial amputation(TTA) and knee disarticulation(KD), and above-knee amputation (AKA) consists of hip disarticulation (HD) and transfemoral amputation (TFA).

Only first-time MLEAs are included as index procedures for analysis, defined as the first MLEA that occur for the patient. Revisions or contralateral MLEA are not considered first-time MLEAs.

Charlson Comorbidity index (CCI) was calculated based on data from DNPR. Diagnosis codes in a 10-year period prior to the index amputation were used and weighted to provide the CCI as described by Quan et al. [18].

Marital status was obtained from the Danish Civil Registration System [13].

The definition of the comorbidity variables for diabetes, cardiovascular disease(CVD), renal insufficiency, peripheral arterial disease (PAD), hypertension and dyslipidemia were based on ICD10 codes from DNPR, and for some diagnoses (diabetes, hypertension, dyslipidemia) also ATC codes from DNPD. As an example a patient was categorized with diabetes if one of the following ICD10 codes was registered: E10*, E11*, E13*, E14* or the patient had redeemed two or more anti-diabetic medicine prescriptions with the following ATC codes within five years before the index date: A10A (Insulins and analogs) A10B (Blood Glucose lowering drugs, excl. insulins), A10X (Other drugs used in diabetes). We used the same definision of a prior would as Madsen et al., but it dit not have to be considered chronic [19]. A complete overview of the definition of diagnoses and procedures is provided in the Appendix 1- Supplementary information.

### Statistic analysis

Descriptive statistics of baseline characteristics for each surgical strategy were performed. First-time MLEA rates per 100.000 inhabitants ≥ 18 years were calculated for each year in the study period using the annual number of procedures and the population ≥ 18 years. Incidence rates were estimated for each year and divided in gender and age group (< 50, 50–70, 71–80, 81–90, and > 90), and presented with 95% confidence intervals (CI). To calculate the annual incidence rates per 100,000 inhabitants in the beginning of each year, numbers from Statistics Denmark were used as the denominator, representing the population size at a given year, stratified in gender and age group. Poisson regression was applied to investigate a possible time trend adjusting for gender and age group. The investigated comorbidities were plotted over the study period. Additionally, we created the same plot, stratifying by initial above-knee and below-knee amputations (see supplemental Fig. 1A-1B).

Post hoc analysis of the data includes a sensitivity analysis of the incidence rates, where the denominator was adjusted for those who already had an amputation, as well as logistic regression analysis to explore significant statistical changes over time for the different surgical strategies and comorbidities during the study period. For all time trend analysis the year of surgery was treated as a continuous variable. Changes in the initial level during the study period were assessed individually for each level. We adjusted for comorbidities (diabetes, PAD, CVD, renal insufficiency, hypertension, dyslipidemia, and prior wound), prior surgery (vascular surgery and minor amputations), gender, CCI-group, and age group. The goodness of fit for the logistic models was checked, and found insignificant for TFA and TTA, but significant for KD and HD, properly due to a small number of observations in these groups. We investigated time trends for the comorbidities and prior surgery: diabetes, PAD, CVD, renal insufficiency, hypertension, prior wound dyslipidemia, prior vascular surgery, and prior minor amputation. All analysis were adjusted for gender, initial index level, age group, and CCI-group, in addition to all comorbidities and prior surgery except the one being analysed. The Goodness of fit for each variable was checked and found to be acceptable. The time trend for a high CCI-score, defined as ≥ 3, high age, defined as ≥ 70 years was analysed using a logistic regression model. This model was adjusted for all comorbidity and prior surgery variables, gender, initial index level, age group, and CCI group. However, the high CCI score was not adjusted for CCI group, and high age was not adjusted for age group. Statistical analyses were performed using STATA version 17.0.

## Results

A total of 12,672 patients underwent first-time MLEA between 2010 and 2021. Patient characteristics for the cohort are listed in Table 1. The median age at first-time MLEA was 74.3 IQR (16.5) for the entire cohort. Overall, 60% were males, higher for TTA with 70% males.

**Table 1:** Patient characteristics for first-time major lower extremity amputations between 2010-2021

	Hip Disarticulation	Transfemoral Amputation	Knee disarticulation	Transtibial amputation
N (%)	**128 (1)**	**7**,**590 (59.9)**	**545 (4.3)**	**4**,**409 (34.8)**
**Baseline Characteristics**				
Age, Median (QR)	69.4 (18.2)	76.7 (15.2)	75.0 (19.1)	70.3 (17.7)
Age group				
< 50	15 (11.7)	200 (2.6)	41 (7.5)	407 (9.2)
50–70	52 (40.6)	1,915 (25.2)	163 (29.9)	1,770 (40.1)
71–80	37 (28.9)	2,529 (33.3)	135 (24.8)	1,332 (30.2)
81–90	24 (18.8)#	2,276 (30)	148 (27.2)	752 (17.1)
< 90	*	670 (8.8)	58 (10.6)	148 (3.4)
Male gender	70 (54.7)	4,174 (55.0)	343 (62.9)	3,103 (70.4)
Married	79 (61.7)	4,179 (55.1)	297 (54.5)	2,547 (57.8)
Trauma associated	10 (7.8)	588 (7.7)	58 (10.6)	415 (9.4)
Sarkoma associated	23 (18.0)	462 (6.1)	29 (5.3)	239 (5.4)
**Comorbidity**				
Renal insufficiency	15 (11.7)	1,057 (13.9)	61 (11.2)	834 (18.9)
CVD	28 (21.9)	2,857 (37.7)	203 (37.2)	1,714 (38.9)
Diabetes	25 (19.5)	2,723 (35.9)	232 (42.6)	2,594 (58.8)
PAD	39 (30.5)	6,106 (80.5)	416 (76.3)	3,368 (76.4)
Hypertension	90 (70.3)	6,587 (86.8)	448 (82.2)	3,705 (84.0)
Dyslipidemia	54 (42.2)	4,560 (60.1)	293 (53.8)	2,881 (65.3)
Prior wound	19 (14.8)	3,463 (45.6)	297 (54.5)	2,382 (54.0)
**Prior Surgery**				
Revascularization	33 (25.8)	2,848 (37.5)	181 (33.2)	1,857 (42.1)
Minor amputation	*	870 (11.5)	103 (18.9)	1,574 (35.7)
**CCI Group**				
0	34 (26.6)	2,327 (30.7)	181 (33.2)	958 (21.7)
1–2	51 (39.8)	3.009 (39.7)	200 (36.7)	2,127 (48.2)
>= 3	43 (33.6)	2,251 (29.7)	164 (30.1)	1,324 (30.0)

Values in count and (%) if not otherwise noted

PAD: Peripheral Arterial Disease

CVD: Cardiovascular disease

CCI: Charlson Comorbidity Index

# includes the patients > 90 in this group

*exact value under 5– not visible due to Danish law regulation

### Incidence rates

**Fig. 2:**
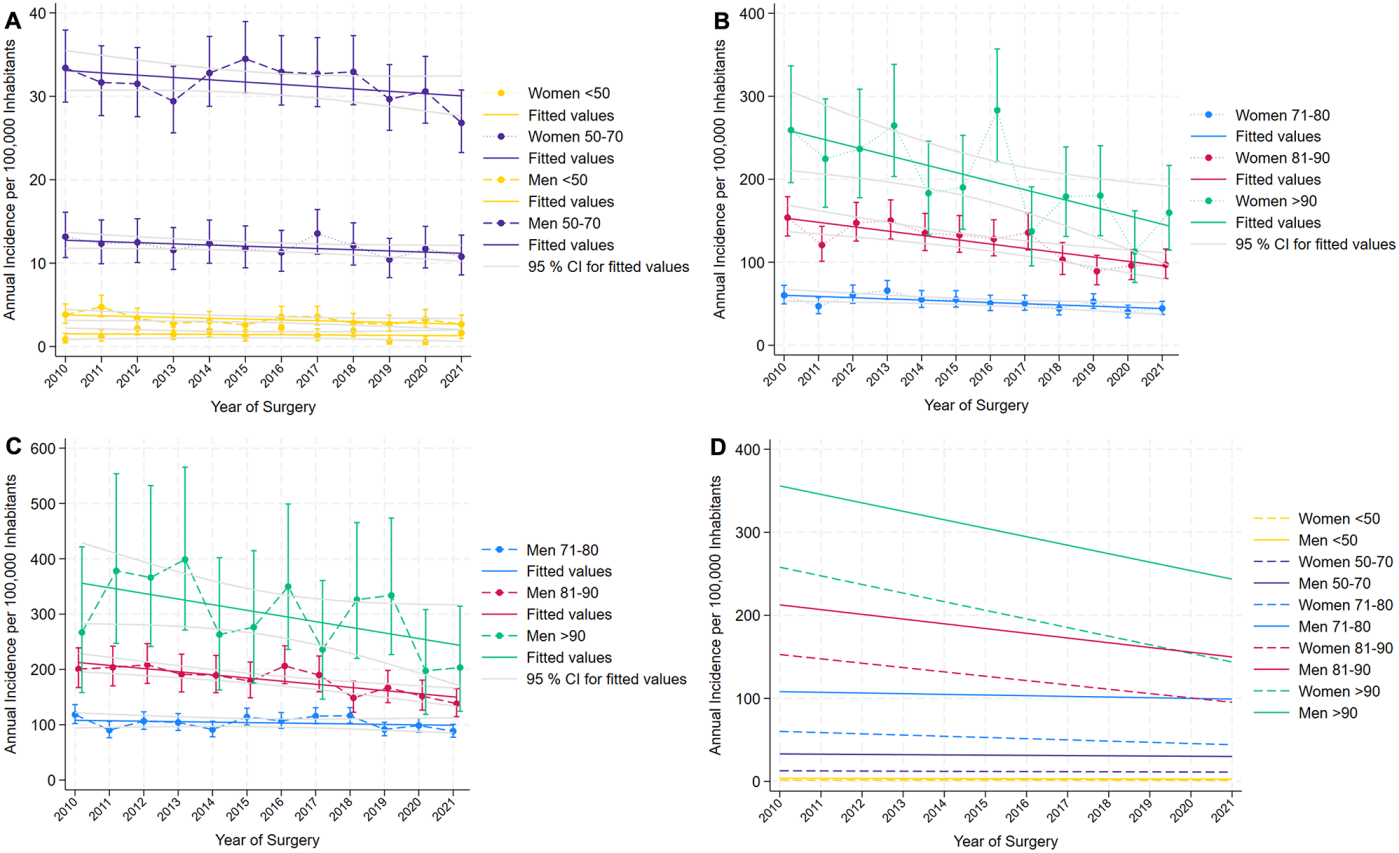
Incidence rates for first-time major lower extremity amputation per 100,000 inhabitants, stratified by age and gender between 2010–2021 (**A-D**). (**A**) Annual incidence rates per 100,000 inhabitants for the age groups < 50 and 50–70, both men and women. (**B**) Annual incidence rates per 100,000 inhabitants for the age groups 71–80, 81–90 and > 90 for women. (**C**) Annual incidence rates per 100,000 inhabitants for the age groups 71–80, 81–90 and > 90 for men. (**D**) Fitted values of annual incidence rates per 100,000 inhabitants for all age groups and both genders

The overall national incidence decreased by 0.8% (95% CI 0.3–1.3, *p* = 0.002) per year unadjusted, and 2.3% (95% CI 1.8–2.8, p = < 0.001) when adjusting for age group and gender. This corresponds to a decrease in the unadjusted rates of 24.7/100,000 inhabitants in the beginning of the year in 2010 to 21.3/100,000 inhabitants in the beginning of the year in 2021 (Table 2). The incidence rates were declining in the study period (Fig. 2A-D). We conducted a sensitivity analysis were we excluded those who already had an amputation and no change in the results was observed. Full overview of calculated incidence rates and sensitivity analysis excluding cases already amputated can be found in Appendix 2- Supplementary Table 1.

**Table 2:** Annual incidence rate of first-time major lower extremity amputations per 100,000 inhabitants in 2010 and 2021

			2010			2021				
			Rate (per 100,000 inhabitants)		95% CI	Rate (per 100,000 inhabitants)			95% CI	
Total national incidence of first-time MLEA in 2010 and 2021 in persons ≥ 18 years			24.7		23.2–26.2	21.2			20.0-22.6	
Male			29.1		26.9–31.5	26.2			24.1–28.4	
Female			20.3		18.5–22.3	16.5			14.9–18.2	
**Incidence stratified by gender and age group**										
	2010				2021					
	Male		Female		Male		Female			
**Age group**	**Rate (per 100**,**000 inhabitants)**	**95% CI**	**Rate (per 100**,**000 inhabitants)**	**95% CI**	**Rate (per 100**,**000 inhabitants)**		**95% CI**	**Rate (per 100**,**000 inhabitants)**		**95% CI**
< 50 years	3.8	2.8–5.1	0.9	0.4–1.6	2.7		1.8–3.8	1.6		1.0-2.6
50–70 years	33.4	29.3–37.9	13.2	10.7–16.1	26.8		23.3–30.8	10.8		8.8–13.4
71–80 years	118.4	102.2-136.4	60.2	49.8–72.2	88.6		77.5-100.8	44.5		37.1–52.9
81–90 years	200.8	167.3–239.0	153.9	131.6–179.0	138.2		114.7-165.3	97.0		80.3–116.0
> 90 years	266.9	158.3-421.6	259.3	195.9-336.6	203.6		124.4-314.3	159.8		114.7-216.7

### Surgical strategies

**Fig. 3:**
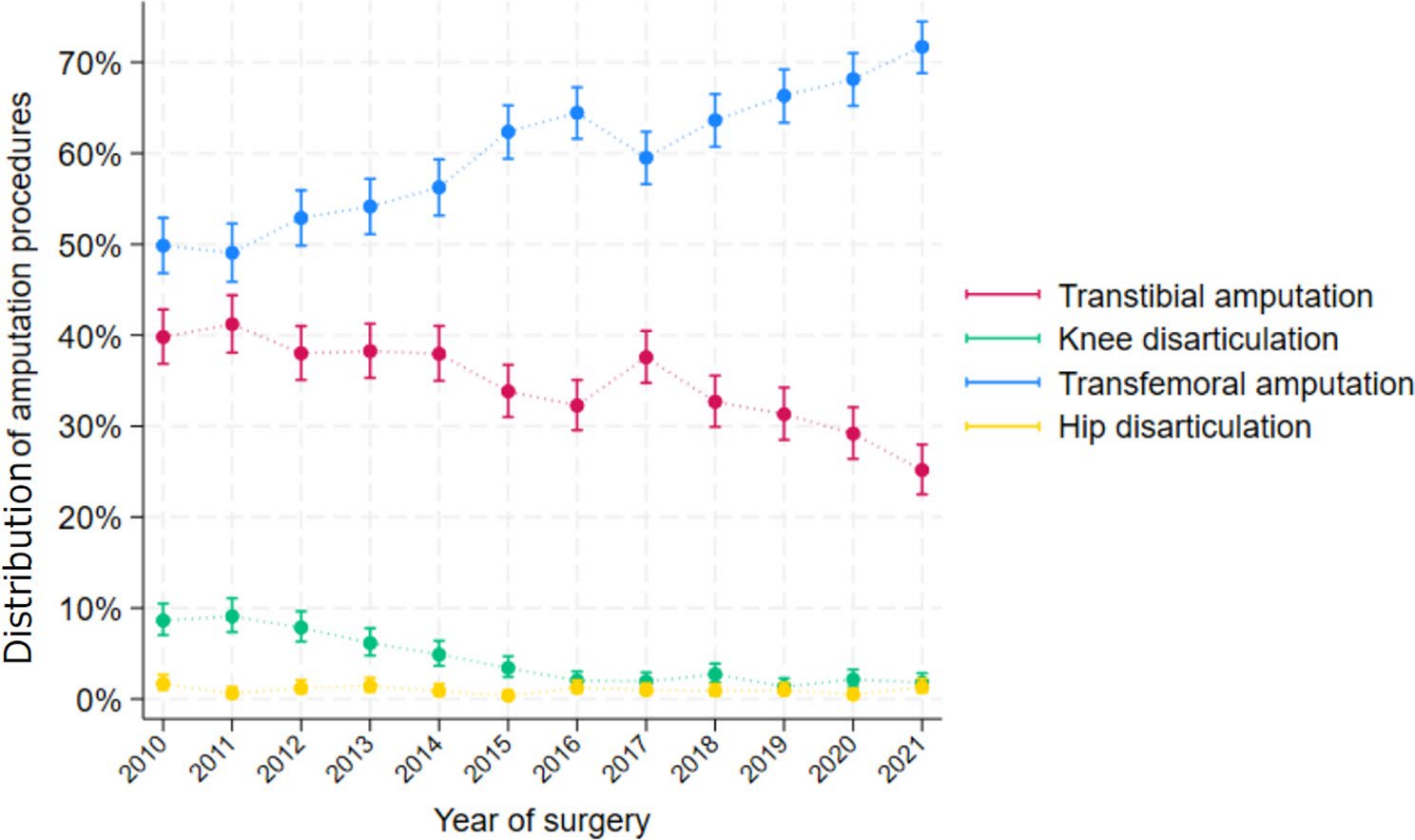
Trend in specific surgical strategies for first-time major lower extremity amputations from 2010–2021

The total number of first-time MLEAs in the study period was stable, ranging from 968 to1139 procedures annually (Fig. 3). The frequency of initial TFA increased while initial KD and initial TTA decreased during the study period. The amount of initial HD procedures were stable over time. When testing for time trends TFA had a significant increase at 10.7% /year CI (9.6–11.9), while TTA and KD had a significant decrease at -7.3%/year CI(-8.4 - -6.1) and − 19.2 (-22- − 16.3) respectively (Table 3).

**Table 3:** Time trends by logistic regression, in crude and adjusted results

		Crude results		Adjusted results*	
		P-value	Coefficient with 95% CI	P-value	Coefficient with 95% CI
Surgical strategies	Hip Disarticulation	0,219	-3.2 (-8.3-1.9)	0,07	-4.8 (-10.5-0.4)
	Transfemoral Amputation	< 0.001	8,3 (7,2–9,3)	< 0.001	10.7 (9.6–11.9)
	Knee disarticulation	< 0.001	-19.2 (-22.1–16,5)	< 0.001	-19.2 (-22.0- -16.3)
	Transtibial Amputation	< 0.001	-5.3 (-6.3–4.2)	< 0.001	-7.3 (-8,4- -6,1)
Comorbidities	Renal insuffiency	0,005	2 (0.6–3.4)	0,027	1.8 (0.2–3.3)
	Cardiovascular disease	0,01	-1.3 (-2.4–0,3)	< 0.001	-3.4 (-4.6–-2.1)
	Diabetes	0,016	1.2 (0,2-2.3)	0,964	0.02 (-1.3-1.2)
	Peripheral arterial disease	< 0.001	-6.1 (-7.3–4.9)	< 0.001	-9.3 (-10.8- -7.7)
	Hypertension	0,43	0,5 (-0.9-2)	0,467	-0.7 (-2.41.1)
	Dyslipidemia	< 0.001	4.3 (3.2–5.3)	< 0.001	5.7 (− 4.5–7.1)
	Prior wound	0,022	1,2 (0,2-2.2)	0,001	1.8 (0.7–2.8)
Prior surgery	Prior vascular surgery	0,565	-0,3 (-1,3 − 0,7)	0,326	0.6 (-0.5-1.8)
	Prior minor amputation	0,001	2.1 (0.9–3.4)	< 0.001	4.2 (2.7–5.6)
CCI and age	CCI score ≥ 3	0,123	0,9 (-0,2 − 1,9)	0,331	0.7 (-0.6-1,9)
	Age ≥ 70	0,044	1,1 (0,02-2.1)	0,004	1.8 (0.6–2.9)

*Adjusted analysis were for surgical strategies adjusted for comorbidities (diabetes, PAD, CVD, renal insufficiency, hypertension, dyslipidemia and prior wound), prior surgery (vascular surgery and minor amputations), gender, CCI-group and age group

For comorbidities and prior surgery the analysis were adjusted for initial level, for the other comorbidities than the one in question (diabetes, PAD, CVD, renal insufficiency, hypertension, dyslipidemia and prior wound), prior surgery (vascular surgery and minor amputations), gender, CCI-group and age group

For the CCI score ≥ 3, the analysis were adjusted for initial level, comorbidities (diabetes, PAD, CVD, renal insufficiency, hypertension, dyslipidemia and prior wound), prior surgery (vascular surgery and minor amputations), gender and age group

For the age ≥ 70, the analysis were adjusted for initial level, comorbidities (diabetes, PAD, CVD, renal insufficiency, hypertension, dyslipidemia and prior wound), prior surgery (vascular surgery and minor amputations), gender and CCI group

PAD: Peropheral arterial disease. CVD: Cardiovascular disease. CCI: Charlson Comorbidity Index

### Comorbidity burden

In the entire cohort, 16% had renal insufficiency, 38% had CVD, 44% had diabetes, 78% had peripheral arterial disease, 86% had hypertension, 62% had dyslipidemia and 49% had a prior wound. Furthermore, 64% were > 70 years old at the time of first MLEA, and 30% had a CCI score of ≥ 3.

**Fig. 4:**
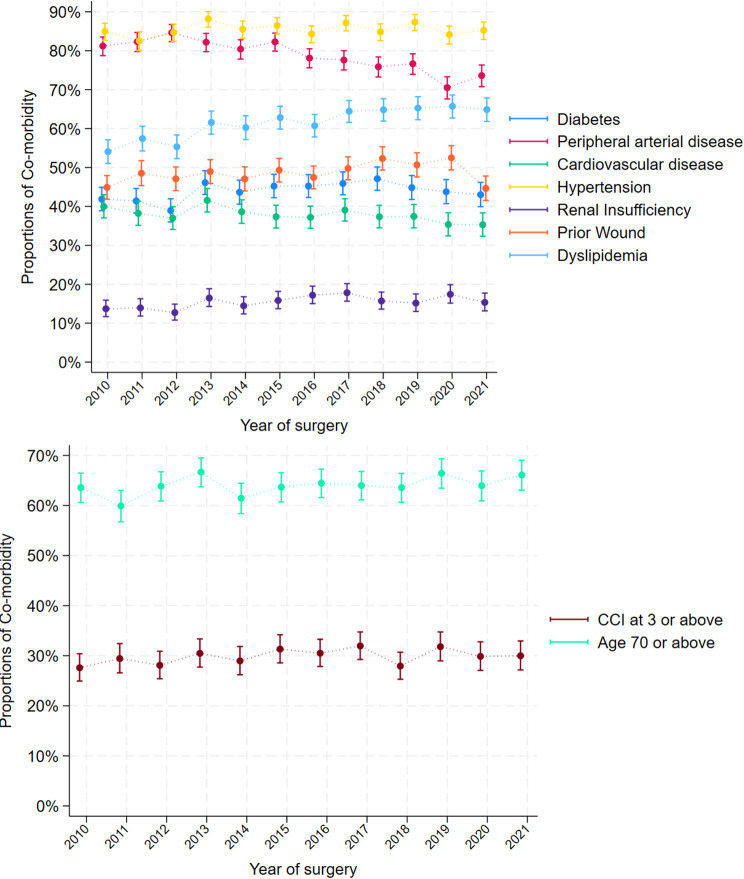
Trend in co-morbidity among first time-major lower extremity amputation patients depending on year af index amputation CCI: Charlson Comorbidity index

The distribution of comorbidities, age ≥ 70, and a CCI score ≥ 3 throughout the study period can be seen in (Fig. 4). The proportion of all the investigated comorbidities, except peripheral arterial disease and dyslipidemia, had overlapping CI’s throughout the study period. When statistically testing for time trends we found that renal insufficiency (1.8%/year CI (0.2–3.3)), dyslipidemia (5.7%/year CI (4.5–7.1)) and prior wound (1.8%/year CI (0.7–2.8)) were slightly increasing in the study period. While CVD (-3.4%/year CI (-4.6- -2.1)) and PAD (-9.3%/year CI (-10.8- -7.7)) were decreasing in the study period. For age at ≥ 70, the time trend was 1.8%/year (0.6–2.9), the time trend for a CCI score ≥ 3 was non-significant (Table 3). For results only for above knee procedures and below knee procedures see supplemental Fig. 1A-B.

### Previous surgical procedures

**Fig. 5:**
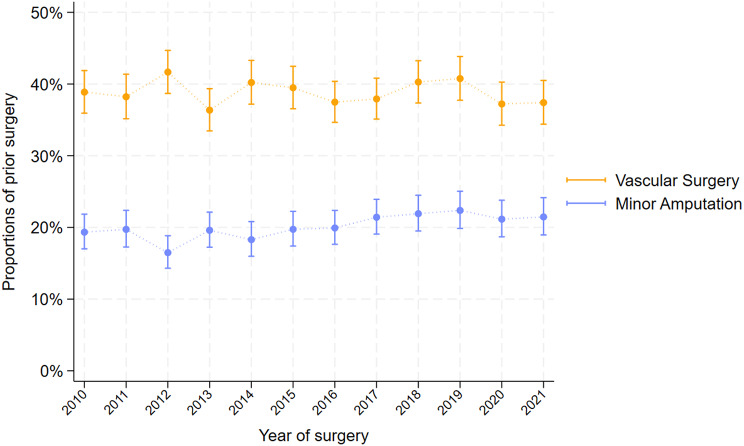
Minor amputation and revascularization surgery before first-time major lower extremity amputation

Overall 20% had a prior minor amputation and 39% had at least 1 vascular surgery before having a major amputation. The proportion of patients with prior relevant surgery is shown in (Fig. 5). When investigating time trends of prior surgery, the trend was significant for minor amputation with 4.2%/year CI (2.7–5.6). For results only for above knee procedures and below knee procedures see Supplemental Fig. 2A-2B.

**Fig. 6:**
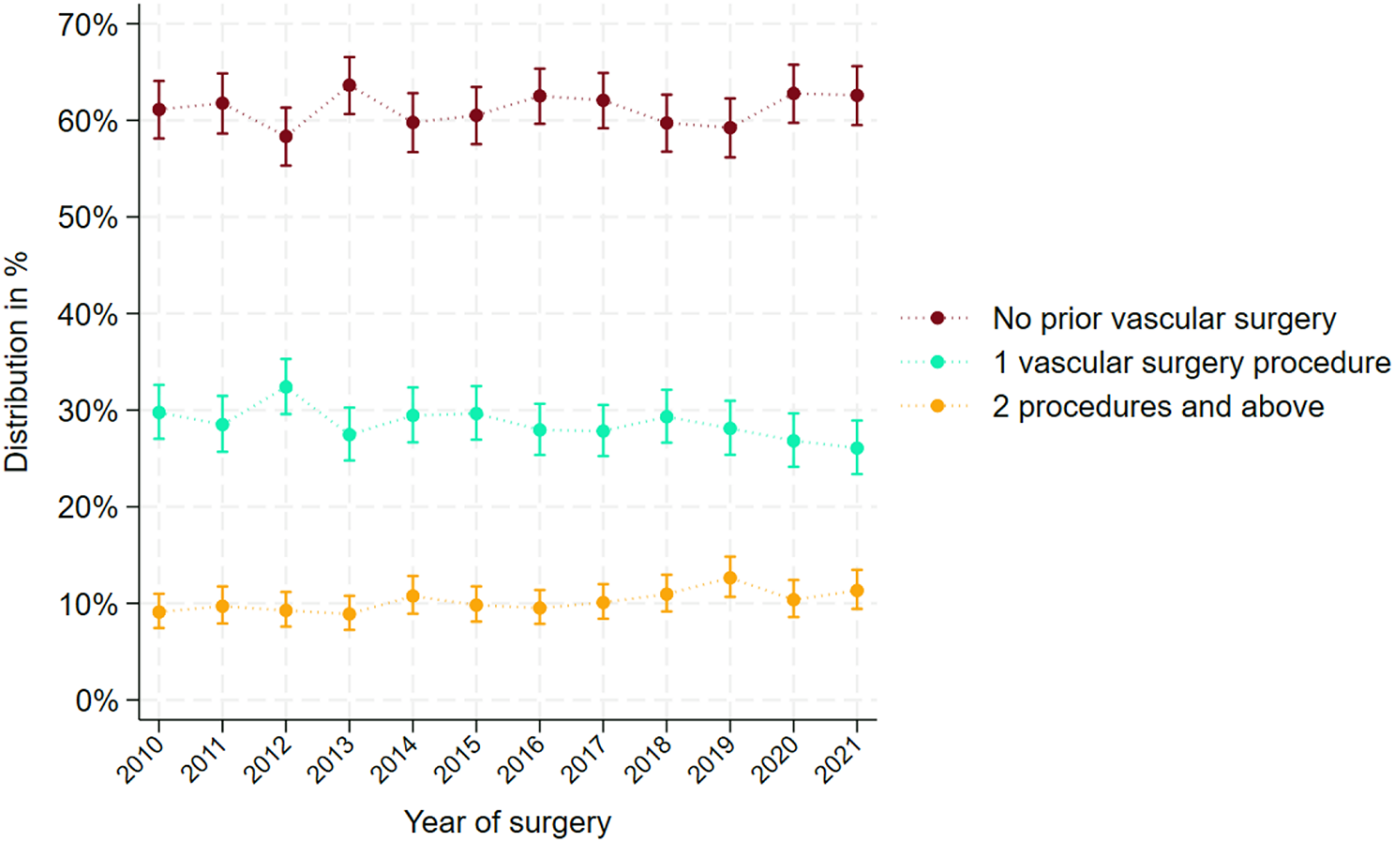
Quantification of revascularization surgeries before first-time major lower extremity amputations

There was no change in the distribution of revascularization procedures, defined as separate dates of revascularization surgery before first-time MLEA throughout the study period (Fig. 6).

## Discussion

In this observational nationwide register study of first-time MLEA, we found a decreasing adjusted incidence and a significant shift in surgical strategy towards more initial TFA procedures and thereby a decrease in initial KD and TTA procedures. We analyzed the comorbidity burden during the study period in order to identify a potential association to this shift in initial level. We found that the comorbidity burden over time were stable, except PAD and dyslipidemia, that decreased. When we investigated time trends, we found significant time trends for renal insufficiency, CVD, PAD, dyslipidemia and prior wound. These changes were mostly subtle, with limited clinical relevance and did not explain the shift in initial level.

### Incidence

This nationwide study support that the adjusted incidence rate of first-time MLEA is decreasing statistical significant since 2010, with a yearly overall adjusted decrease of 2.3% [3, 4, 6, 20, 21]. However, the number of absolute cases was overall constant, and hence MLEA’s are still common surgical procedures. 

Incidence rates adjusted for age and gender reveal major differences in incidences and the decrease is especially mediated by the population aged > 80, while it was constant over time for the younger population.

### Surgical strategies

We found a significant shift towards a more proximal first-time amputation level, with TFA as the most common first time MLEA, and increasing in the study period, while initial KD and initial TTA were decreasing. This rise in initial TFA does not seem to be explained in an associated severely altered comorbidity profile of the MLEA patients.

The decrease in KD was also found in a recent German nationwide study, where KD was reduced considerably from 2015 to 2019 [5]. The KD procedure has been investigated previously in a single-center study from Denmark, finding that a reduction in the frequency of KD led to decreased overall risk of re-amputations [22]. This might explain some of the decrease in KD procedures in this study, but it does not solely explain the shift in surgical strategy and the reduction in TTA procedures.

The choice of TFA instead of TTA could be explained by an expected lower risk of re-amputation despite that the risk of mortality may be higher [9, 16, 17, 22].

It is unknown if this trend in surgical strategies towards first-time MLEA is seen outside Europe as use of surgical strategies are sparsely described, and lack of consensus on definition challenge head-to-head comparison of results [7].

### Comorbidity burden and previous surgical procedures

The high comorbidity burden and unequal distribution of gender in our study was comparable to other studies [5, 8, 9]. In the last decade, there has been an increased focus on prevention, e.g., interdisciplinary diabetic foot ulcer centers, enhanced wound care, and extensive development in vascular surgery. This might explain the overall reduction in incidence rates of MLEA. However, the age and comorbidity burden at first-time MLEA did not increase as expected if the increased focus had prevented or postponed amputations in some of the “healthiest” patients in the potential amputation population. We did however find some significant time trends, but these were subtle with limited clinical relevance, and both increasing a decreasing. We observed no clinical relevant alteration in the comorbidity profile of these patients that could explain the drastic changes in the surgeon’s choice of initial level. We found no increase in revascularization procedures before first-time MLEA, and this could not explain the trend towards a more proximal level [8], and while minor amputation before a MLEA had a significant time trend this was seen in both above and below knee amputations (Supplemental Fig. 1A-B). Despite the rising global incidence of diabetes, the incidence of MLEAs in the diabetic population has been decreasing [2, 23, 24]. In our study, the prevalence of diabetes in first-time MLEA patients was constant during the study period reflecting that the decrease of MLEAs in the diabetic population did not lead to a reduction in the actual number of MLEAs.

MLEA studies typically focus only on patients with PAD and/or diabetes [3, 8, 10, 21, 23–25]. In contrast, we included all first-time MLEA on adult patients to provide an extensive overview of all first-time MLEAs and choice of index amputation level. So far, no international consensus about how to choose the appropriate level of MLEA exists. Often it is the surgeon’s individual assessment that decide the offered index level, and few studies has investigated models for level determination [25, 26]. Our study showed a significant increase of TFAs leading towards a more proximal level for first-time MLEAs. There was no clear association with a rise in age and comorbidities in these patients. This pattern might be present in other countries as well, and reasons for the more proximal index level should be investigated further.

### Strengths and limitations

Data completeness from Danish health registers are high, estimated 99% [27]. The main strength of this study was the inclusion of the whole population of interest, though nationwide registers, thus avoiding selection bias. The data were extracted from high quality administrative registers, which lower the risk of information bias [27].

The registries provide an opportunity to calculate comorbidity distribution and burden e.g. CCI, but it may still be a limitation that it was based on diagnosis codes and not validated with data from patient records. The Danish registers shifted from LPR2 to LPR3 in 2018, which might have resulted in changes. Furthermore, the registers do not contain information on the indication for amputation and the procedure codes for amputations are not yet validated. Data’s completeness and correctness in coding depend on the surgeon to code the procedure correctly, including the site (left/right) [28].

For the incidence rates, we used numbers from Statistics Denmark that in the beginning of each year reports the population. We therefore do not have access to how many in each age group that died each year. This could especially be a limitation in the oldest groups, but we do not expect it to alter with the results of this study.

## Conclusion

We observed a decreasing incidence of first-time MLEA in Denmark and a shift towards increased use of transfemoral amputations as initial MLEA level. Investigation of the comorbidity profile of MLEA patients revealed some time trend changes during the study period, but with limited clinical relevance. Hence, the observed prominent shift towards a more proximal first time amputation level in Denmark did not seem to be associated with an altered comorbidity profile of these patients. Whether the change in surgical strategy is to the benefit of the patients should be investigated further.

## Electronic supplementary material

Below is the link to the electronic supplementary material.

Supplementary Material 1

Supplementary Material 2

## Data Availability

Raw data cannot be shared for privacy reasons.

## Abbreviations

MLEA: Major Lower Extremity Amputation
HD: Hip Disarticulation
TFA: Transfemoral Amputation
KD: Knee Disarticulation
TTA: Transtibial Amputation
ASA: American Society of Anesthesiologists
DNPR: The Danish National Patient Register
DNPD: The Danish National Prescription Database
CCI: Charlson Comorbidity index
BKA: Below Knee Amputation
AKA: Above Knee Amputation
CVD: Cardiovascular disease
PAD: Peripheral arterial disease
CI: Confidence interval
QR: Interquartile range
