# Supplementary material for: Epidemiology of first-time major lower extremity amputations– A Danish Nationwide cohort study from 2010 to 2021
Source: Eur J Epidemiol. 2025 Mar 12;40(3):297–307. doi: 10.1007/s10654-025-01210-3 (PMC12137412; doi:10.1007/s10654-025-01210-3)
Supplement: Supplementary file 1 — Supplementary Material 1 [file 10654_2025_1210_MOESM1_ESM.docx]

# Appendix 1 – Supplementary information

This appendix addresses the definition of variables.

**Amputation codes**

| **Major Amputations** | |
| --- | --- |
| *Above Knee Amputation* | |
| Hip disarticulation | KNFQ09 |
| Transfemoral amputation  Other amputation on the femur/hip | KNFQ19  KNFQ99 |
| *Below Knee Amputation* | |
| Knee disarticulation | KNGQ09 |
| Transtibial amputation  Other amputation on knee/tibia | KNGQ19  KNGQ99 |
| **Prior minor amputation** was defined as one or more registered procedures before index surgery with procedure code KNHQ* despite TUL codes. | |
| **Minor amputations** | |
| Foot amputation | KNHQ10-18, KNHQ99 KNHQ00 |
| Toe amputation | KNHQ02-07 KNHQ14 |

**Revision codes, major amputation**: KNGQ29*, KNGQ39, KNGQ49, KNFQ29*, KNFQ39, KNFQ49

The case was excluded in case of a revision code without a prior primary amputation code. KNGQ99/KNFQ99 (43 cases) are grouped as Transfemoral amputation (KNFQ19) or Transtibial amputation (KNGQ19), respectively.

**Trauma associated:**

An amputation was classified as trauma associated if one of the following ICD10 codes were present: DS980 (traumatic amputation, foot), DS88* (traumatic amputation, knee), DS78* (traumatic amputation, hip), DT136 (traumatic amputation, lower extremity, unspecified), DT05, DT053-6, DT058-9 (traumatic amputation, in combination), DT036 (sequelae after traumatic amputation, lower extremity).

**Sarcoma associated:**

An amputation was classified as trauma associated if one of the following diagnosis codes were present

C492* (cancer, soft tissue, lower extremity), C402*-3*(cancer, bone or cartilage, lower extremity) C408-9(cancer, bone or cartilage, unspecified).

**Prior Revascularization procedure:**

Prior revascularization procedure was defined as one or more registered revascularization procedures before index: KPDA*, KPDC*, KPDE*, KPDF*, KPDH*, KPDN*, KPDP*, KPDQ*, KPDT10, KPDT30, KPDU70, KPDU74, KPDU82-84, KPDU87, KPDU99, KPEA*, KPEC*, KPEE*, KPEF*, KPEH*, KPEN*, KPEP*, KPEQ* KPET*, KPEU74, KPEU82-84, KPEU87, KPEU99, KPFE*, KPFH*, KPFN*, KPFP*, KPFT*, KPFQ*, KPFU74, KPFU82-84, KPFU87, KPFU99, KPGH*, KPGU74, KPGU83-84, KPGU87, KPGU99, KPGW99.

The number of revascularization procedures was defined as separate days of surgery.

**Definition for diagnosis, ICD10:**

**Diabetes:** A patient was categorized with diabetes if one of the following ICD10 codes were registered: E10*, E11*, E13*, E14* or the patient has redeemed two or more anti-diabetic medicine prescriptions in the same ATC group with the following ATC codes five years before the index date: A10A (Insulins and analogs) A10B (Blood Glucose lowering drugs, excl. insulins), A10X (Other drugs used in diabetes).

**Hypertension:** A patient was categorized with hypertension if one of the following ICD10 codes I10*-I15* were registered or the patient has redeemed two or more prescriptions in the same ATC group five years before the index date: antihypertensives (C02-C03, C07- C09).

**Dyslipidemia:** A patient is categorized with Dyslipidemia if the the ICD10-code: E78 was registered, or the patient had redeemed two or more prescriptions in the same ATC group five years before the index date: C10.

**Cardiovascular disease (CVD):** A patient was categorized with CVD if one of the following ICD10 codes were registered: I20-I25* (angina, myocardial infarction, and ischemic coronary disease), I50* (incompensatio cordis), I63-I67 (cerebral infarction, occlusion of vertebral and cerebral arteries) or G45-G46 (transient ischemic attack, TCI).

**Renal insufficiency:** A patient was categorized with Renal insufficiency if one of the following ICD10 codes were registered: N18*, (Chronic kidney disease) or N19* (Unspecified kidney failure).

**Arteriosclerosis/PAD:** Arteriosclerosis/Peripheral arterial disease (PAD) was defined as one or more of the following ICD10 codes: I70, I702, I709, I739A, I739C or I743

**Prior wound:** Prior wound was defined as one or more of the following ICD10 codes registered:

I830*, I822* (Varicose ulcer, lower extremity), L024A,B,F,I,J,M (abscess, lower extremity), L889 (Pyoderma gangrenosum). L97* (Ulcer, lower extremity) T130-31 (injury or open wound, lower extremity), T138-9 (other lesion, lower extremity), T930 (sequelae of wound, lower extremity), S81*, S89*, S91* (open wound, unspecific injuries, lower extremity) (1)

1. Madsen UR, Hyldig N, Juel K. Outcomes in patients with chronic leg wounds in Denmark: A nationwide register-based cohort study. Int Wound J. 2022;19(1):156-68.
