# Supplementary material for: Epidemiology of first-time major lower extremity amputations– A Danish Nationwide cohort study from 2010 to 2021
Source: Eur J Epidemiol. 2025 Mar 12;40(3):297–307. doi: 10.1007/s10654-025-01210-3 (PMC12137412; doi:10.1007/s10654-025-01210-3)

# Appendix 2 - Supplementary tables and figures

| Supplementary table 1 – Annual Incidence rates with 95% CI, stratified by gender and age categories | | |
| --- | --- | --- |
|  | **Female** | **Male** |
| 2010 |  |  |
| <50 | 0.9 (0.4; 1.6) | 3.8 (2.8; 5.1) |
| 50-70 | 13.2 (10.7; 16.1) | 33.4 (29.3; 37.9) |
| 71-80 | 60.2 (49.8; 72.2) | 118.4 (102.2; 136.4) |
| 81-90 | 153.9 (131.6; 178.9) | 200.8 (167.3; 239.1) |
| >90 | 259.3 (195.9; 336.6) | 266.9 (158.3; 421.6) |
| 2011 |  |  |
| <50 | 1.2 (0.7; 2.0) | 4.7 (3.6; 6.2) |
| 50-70 | 12.3 (9.9; 15.2) | 31.7 (27.7; 36.0) |
| 71-80 | 47.0 (38.0; 57.6) | 90.4 (76.5; 106.1) |
| 81-90 | 120.8 (101.1; 143.2) | 203.8 (170.3; 242.0) |
| >90 | 224.7 (166.3; 296.9) | 378.3 (247.3; 553.8) |
| 2012 |  |  |
| <50 | 2.2 (1.4; 3.2) | 3.4 (2.4; 4.6) |
| 50-70 | 12.5 (10.1; 15.3) | 31.5 (27.6; 35.9) |
| 71-80 | 60.8 (50.5; 72.5) | 106.7 (91.8; 123.4) |
| 81-90 | 147.5 (125.5; 172.1) | 208.5 (174.8; 246.7) |
| >90 | 236.6 (177.8; 308.5) | 366.2 (241.4; 532.3) |
| 2013 |  |  |
| <50 | 1.5 (0.9; 2.3) | 2.8 (1.9; 3.9) |
| 50-70 | 11.6 (9.2; 14.3) | 29.4 (25.6; 33.6) |
| 71-80 | 65.9 (55.4; 78.0) | 104.2 (89.7; 120.3) |
| 81-90 | 150.1 (127.9; 175.1) | 191.2 (159.3; 227.6) |
| >90 | 264.6 (203.4; 338.4) | 398.8 (271.1; 565.5) |
| 2014 |  |  |
| <50 | 1.9 (1.2; 2.9) | 3.0 (2.1; 4.2) |
| 50-70 | 12.4 (10.0; 15.2) | 32.8 (28.8; 37.2) |
| 71-80 | 55.1 (45.6; 65.9) | 91.6 (78.3; 106.3) |
| 81-90 | 135.1 (114.1; 158.9) | 189.5 (158.0; 225.4) |
| >90 | 183.2 (133.1; 245.9) | 263.2 (163.0; 402.0) |
| 2015 |  |  |
| <50 | 1.2 (0.7; 2.0) | 2.6 (1.8; 3.7) |
| 50-70 | 11.8 (9.4; 14.5) | 34.5 (30.4; 39.0) |
| 71-80 | 55.2 (46.0; 65.8) | 113.7 (99.3; 129.6) |
| 81-90 | 132.7 (112.0; 156.3) | 178.9 (148.7; 213.4) |
| >90 | 190.2 (139.8; 252.8) | 276.5 (175.4; 414.6) |
| 2016 |  |  |
| <50 | 2.2 (1.5; 3.3) | 3.6 (2.6; 4.8) |
| 50-70 | 11.3 (9.0; 13.9) | 32.9 (28.9; 37.3) |
| 71-80 | 50.4 (41.8; 60.2) | 107.2 (93.6; 122.1) |
| 81-90 | 128.1 (107.8; 151.1) | 206.4 (174.3; 242.7) |
| >90 | 283.1 (221.2; 357.0) | 349.9 (236.2; 499.1) |
| 2017 |  |  |
| <50 | 1.3 (0.7; 2.1) | 3.6 (2.6; 4.8) |
| 50-70 | 13.6 (11.1; 16.4) | 32.7 (28.8; 37.0) |
| 71-80 | 50.6 (42.2; 60.2) | 115.7 (102.0; 130.7) |
| 81-90 | 135.7 (114.9; 159.2) | 190.0 (159.7; 224.3) |
| >90 | 137.2 (95.6; 190.8) | 236.1 (146.2; 360.7) |
| 2018 |  |  |
| <50 | 1.9 (1.2; 2.9) | 2.8 (2.0; 4.0) |
| 50-70 | 12.1 (9.8; 14.8) | 32.9 (29.0; 37.3) |
| 71-80 | 44.3 (36.6; 53.1) | 116.3 (103.0; 130.9) |
| 81-90 | 103.2 (85.3; 123.7) | 148.8 (122.6; 178.8) |
| >90 | 179.1 (131.2; 238.9) | 326.2 (220.2; 465.3) |
| 2019 |  |  |
| <50 | 0.7 (0.3; 1.3) | 2.7 (1.8; 3.8) |
| 50-70 | 10.4 (8.3; 13.0) | 29.7 (25.9; 33.8) |
| 71-80 | 52.4 (44.2; 61.6) | 91.7 (80.2; 104.5) |
| 81-90 | 88.4 (72.1; 107.3) | 167.2 (139.9; 198.2) |
| >90 | 180.3 (132.0; 240.4) | 333.9 (227.0; 473.6) |
| 2020 |  |  |
| <50 | 0.6 (0.2; 1.2) | 3.3 (2.3; 4.4) |
| 50-70 | 11.7 (9.4; 14.4) | 30.6 (26.8; 34.8) |
| 71-80 | 40.2 (33.2; 48.3) | 98.7 (86.9; 111.7) |
| 81-90 | 95.9 (79.2; 115.3) | 151.9 (126.5; 180.8) |
| >90 | 112.9 (75.6; 162.1) | 197.5 (119.0; 308.3) |
| 2021 |  |  |
| <50 | 1.6 (1.0; 2.5) | 2.7 (1.8; 3.8) |
| 50-70 | 10.8 (8.6; 13.4) | 26.8 (23.3; 30.8) |
| 71-80 | 44.5 (37.1; 52.8) | 88.6 (77.5; 100.8) |
| 81-90 | 96.9 (80.3; 116.0) | 138.2 (114.6; 165.3) |
| >90 | 159.8 (114.7; 216.7) | 203.6 (124.4; 314.3) |

## **Supplementary table 2**

| Supplementary table 1 – Annual Incidence rates with 95% CI, stratified by gender and age categories | | |
| --- | --- | --- |
|  | **Female** | **Male** |
| 2010 |  |  |
| <50 | 0.9 (0.4-1.6) | 3.8 (2.8-5.1) |
| 50-70 | 13.2 (10.7-16.1) | 33.4 (29.3-37.9) |
| 71-80 | 60.3 (49.8-72.2) | 118.6 (102.3-136.6) |
| 81-90 | 154.1 (131.8-179.2) | 201.2 (167.7-239.5) |
| >90 | 260.0 (196.4-337.5) | 267.7 (158.7-422.7) |
| 2011 |  |  |
| <50 | 1.2 (0.7-2.0) | 4.7 (3.6-6.2) |
| 50-70 | 12.3 (9.9-15.2) | 31.7 (27.7-36.1) |
| 71-80 | 47.1 (38.0-57.7) | 90.5 (76.6-106.2) |
| 81-90 | 120.9 (101.2-143.4) | 204.3 (170.7-242.5) |
| >90 | 225.2 (166.6-297.6) | 379.7 (248.2-555.9) |
| 2012 |  |  |
| <50 | 2.2 (1.4-3.2) | 3.4 (2.4-4.6) |
| 50-70 | 12.5 (10.1-15.3) | 31.5 (27.6-35.9) |
| 71-80 | 60.8 (50.5-72.6) | 106.9 (91.9-123.5) |
| 81-90 | 147.7 (125.7-172.4) | 208.9 (175.2-247.2) |
| >90 | 237.1 (178.2-309.3) | 367.5 (242.3-534.2) |
| 2013 |  |  |
| <50 | 1.5 (0.9-2.3) | 2.8 (1.9-3.9) |
| 50-70 | 11.6 (9.2-14.3) | 29.4 (25.6-33.6) |
| 71-80 | 66.0 (55.4-78.0) | 104.3 (89.8-120.5) |
| 81-90 | 150.4 (128.1-175.4) | 191.6 (159.6-228.0) |
| >90 | 265.3 (203.9-339.3) | 400.4 (272.2-567.8) |
| 2014 |  |  |
| <50 | 1.9 (1.2-2.9) | 3.0 (2.1-4.2) |
| 50-70 | 12.4 (10.0-15.2) | 32.8 (28.8-37.2) |
| 71-80 | 55.1 (45.7-66.0) | 91.6 (78.4-106.4) |
| 81-90 | 135.3 (114.2-159.1) | 189.8 (158.3-225.8) |
| >90 | 183.5 (133.4-246.3) | 263.9 (163.4-403.0) |
| 2015 |  |  |
| <50 | 1.2 (0.7-2.0) | 2.6 (1.8-3.7) |
| 50-70 | 11.8 (9.4-14.5) | 34.5 (30.4-39.0) |
| 71-80 | 55.3 (46.0-65.9) | 114.3 (99.9-130.2) |
| 81-90 | 132.9 (112.1-156.5) | 179.2 (148.9-213.8) |
| >90 | 190.5 (140.0-253.3) | 277.3 (175.8-415.8) |
| 2016 |  |  |
| <50 | 2.2 (1.5-3.3) | 3.6 (2.6-4.8) |
| 50-70 | 11.3 (9.0-13.9) | 32.9 (29.0-37.3) |
| 71-80 | 50.4 (41.8-60.3) | 107.3 (93.7-122.3) |
| 81-90 | 128.2 (107.9-151.3) | 206.8 (174.7-243.2) |
| >90 | 283.9 (221.8-358.0) | 351.1 (237.0-500.8) |
| 2017 |  |  |
| <50 | 1.3 (0.7-2.1) | 3.6 (2.6-4.8) |
| 50-70 | 13.6 (11.1-16.4) | 32.7 (28.8-37.0) |
| 71-80 | 50.6 (42.2-60.2) | 115.8 (102.1-130.8) |
| 81-90 | 135.9 (115.0-159.4) | 190.4 (160.1-224.7) |
| >90 | 137.4 (95.7-191.1) | 236.7 (146.6-361.6) |
| 2018 |  |  |
| <50 | 1.9 (1.2-2.9) | 2.8 (2.0-4.0) |
| 50-70 | 12.1 (9.8-14.8) | 32.9 (29.0-37.3) |
| 71-80 | 44.3 (36.7-53.1) | 116.5 (103.1-131.1) |
| 81-90 | 103.3 (85.3-123.9) | 149.0 (122.8-179.1) |
| >90 | 179.4 (131.4-239.3) | 327.3 (220.9-466.9) |
| 2019 |  |  |
| <50 | 0.7 (0.3-1.3) | 2.7 (1.8-3.8) |
| 50-70 | 10.4 (8.3-13.0) | 29.7 (25.9-33.8) |
| 71-80 | 52.7 (44.5-62.1) | 91.8 (80.2-104.6) |
| 81-90 | 89.4 (72.9-108.4) | 167.5 (140.1-198.5) |
| >90 | 180.6 (132.2-240.8) | 335.0 (227.7-475.2) |
| 2020 |  |  |
| <50 | 0.6 (0.2-1.2) | 3.3 (2.3-4.4) |
| 50-70 | 11.7 (9.4-14.4) | 30.6 (26.8-34.8) |
| 71-80 | 40.2 (33.2-48.3) | 98.8 (87.0-111.8) |
| 81-90 | 96.0 (79.2-115.4) | 152.1 (126.7-181.1) |
| >90 | 113.0 (75.7-162.3) | 197.9 (119.2-308.9) |
| 2021 |  |  |
| <50 | 1.6 (1.0-2.5) | 2.7 (1.8-3.8) |
| 50-70 | 10.8 (8.6-13.4) | 26.8 (23.3-30.8) |
| 71-80 | 44.5 (37.2-52.9) | 88.6 (77.6-100.9) |
| 81-90 | 97.0 (80.4-116.1) | 138.4 (114.8-165.5) |
| >90 | 160.0 (114.8-217.0) | 204.0 (124.7-314.9) |

# Supplemental Figures

# Sup. Figure 1A

Comorbidty for Above knee amputation


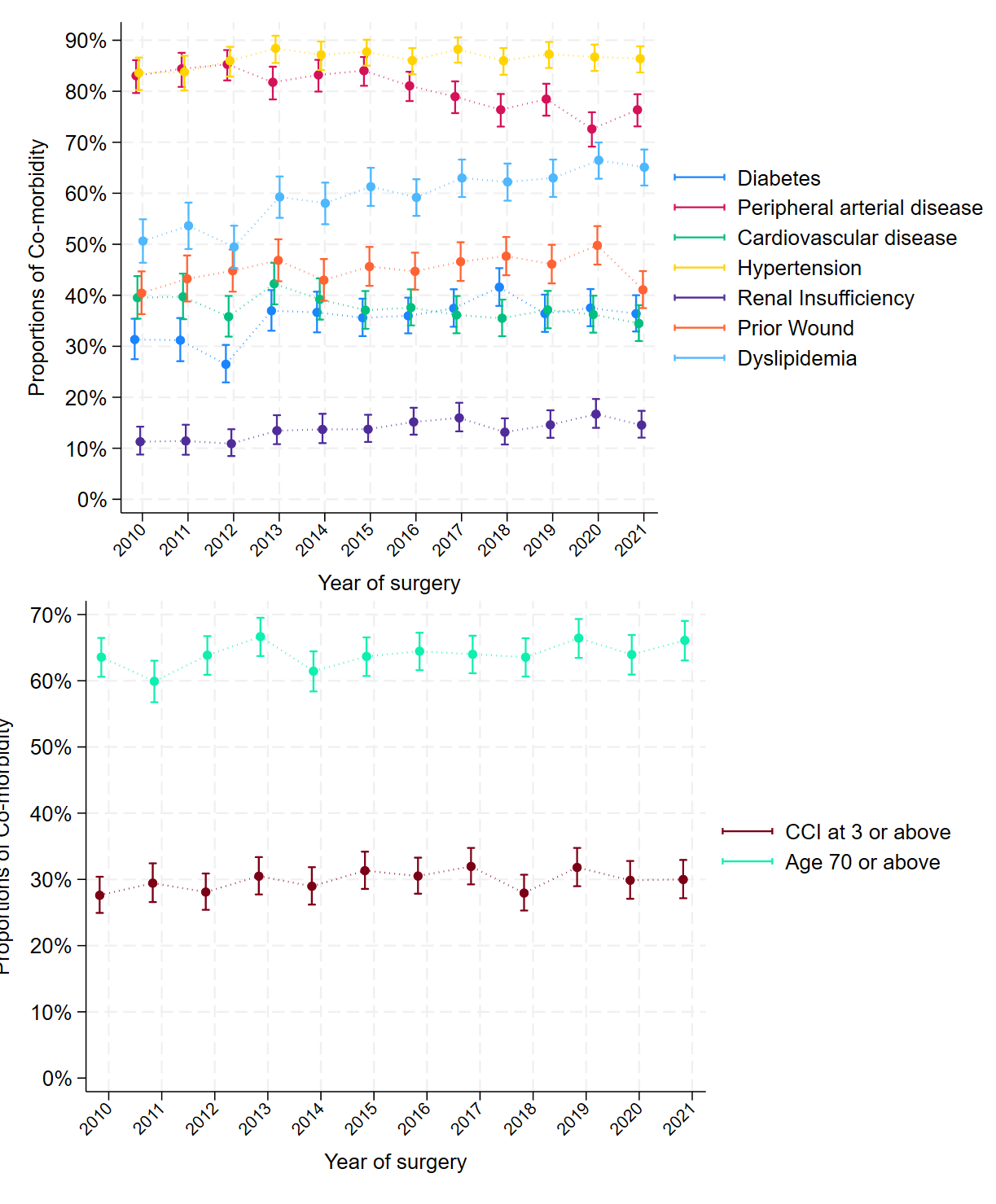


Sup. Figure 1B

Comorbidity for below knee amputation


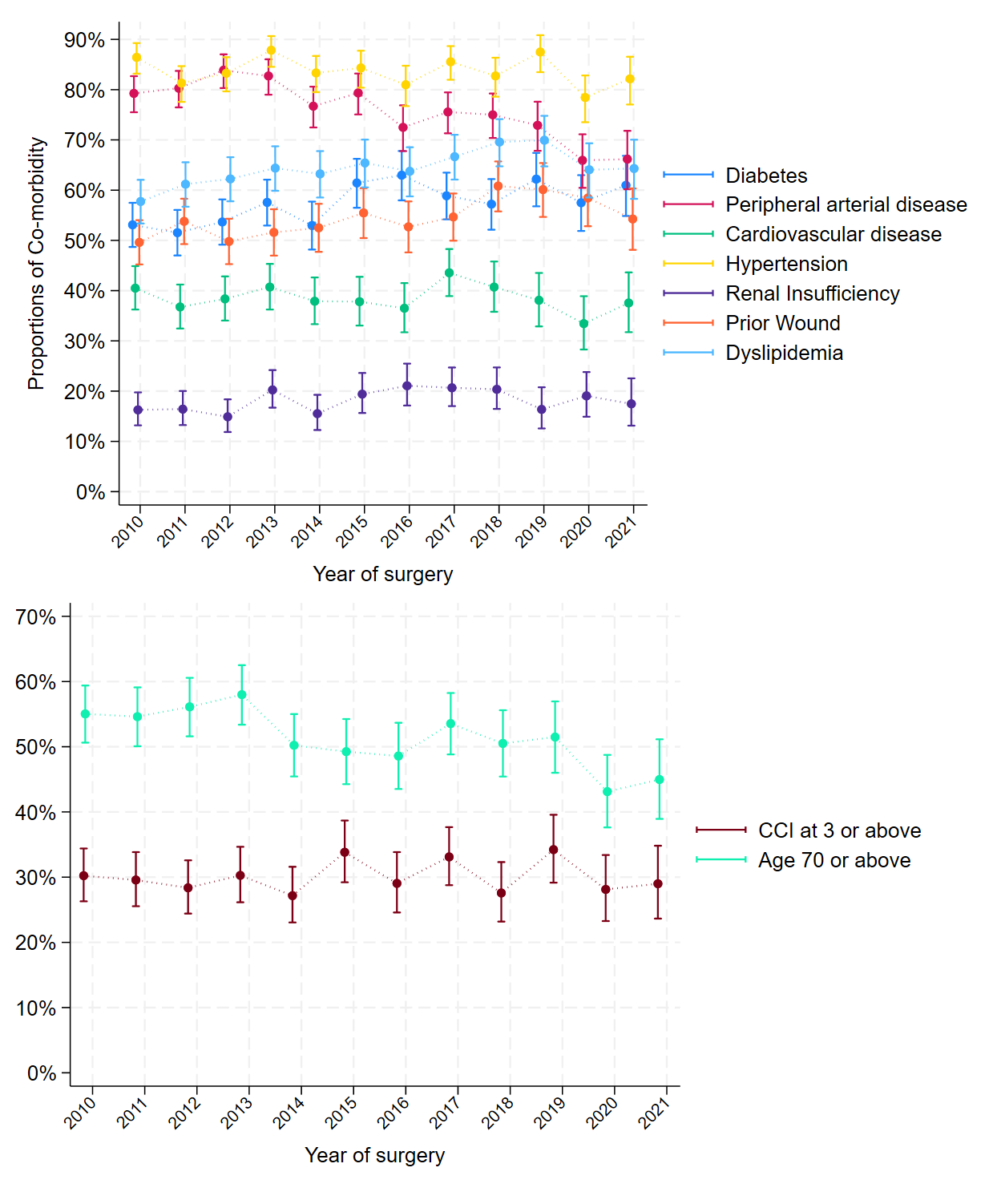


Supplemental Figure 2APrior surgery for Above knee amputations


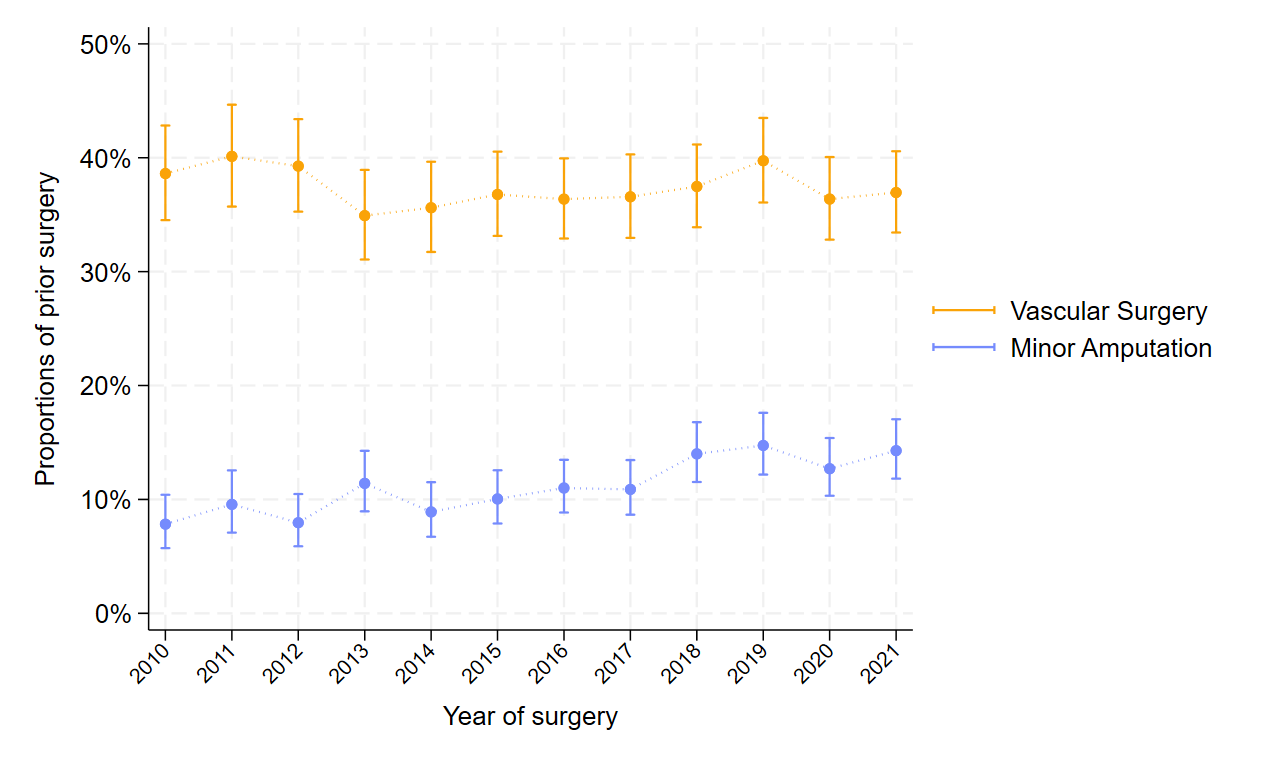


Supplemental Figure 2B Prior surgery for below knee amputations


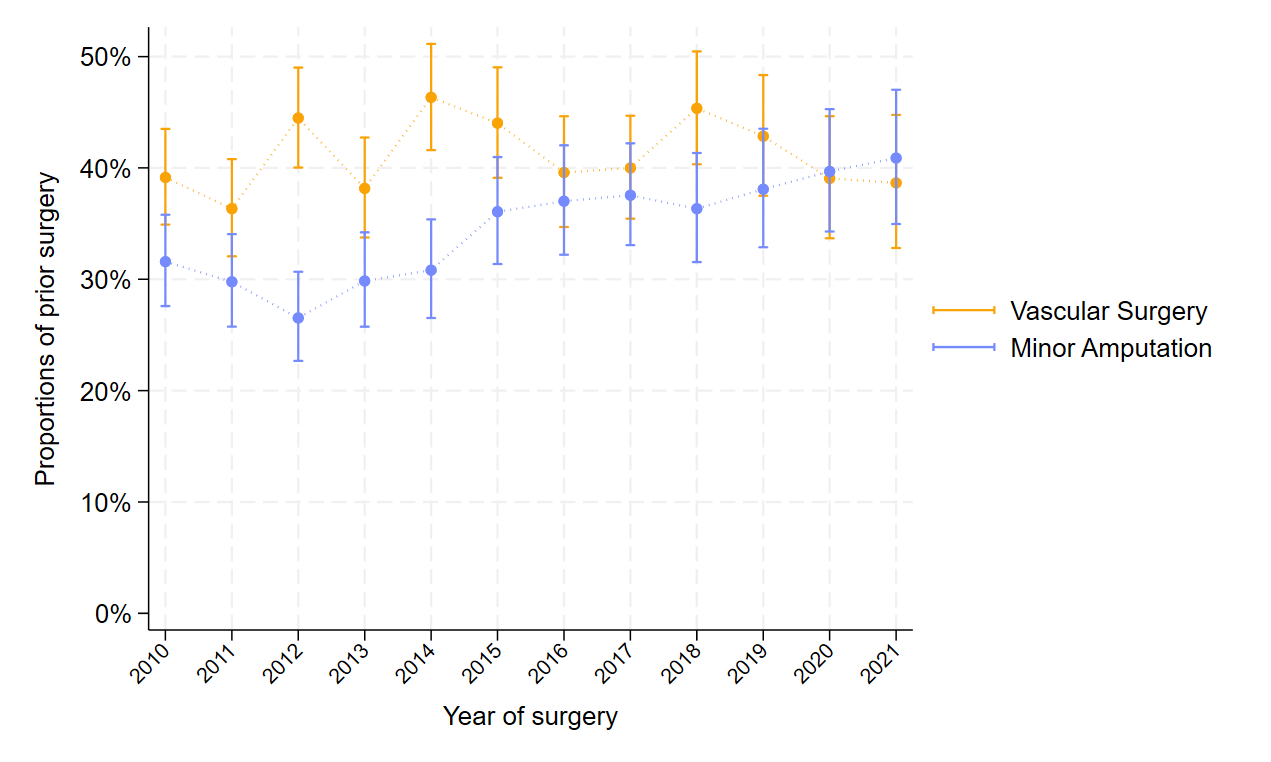

Supplement: Supplementary file 2 — Supplementary Material 2 [file 10654_2025_1210_MOESM2_ESM.docx]
